# Laboratory information systems for research management in biology

**DOI:** 10.18699/VJGB-23-104

**Published:** 2023-12

**Authors:** A.M. Mukhin, F.V. Kazantsev, S.A. Lashin

**Affiliations:** Institute of Cytology and Genetics of the Siberian Branch of the Russian Academy of Sciences, Novosibirsk, Russia Kurchatov Genomic Center of ICG SB RAS, Novosibirsk, Russia Novosibirsk State University, Novosibirsk, Russia; Institute of Cytology and Genetics of the Siberian Branch of the Russian Academy of Sciences, Novosibirsk, Russia Kurchatov Genomic Center of ICG SB RAS, Novosibirsk, Russia Novosibirsk State University, Novosibirsk, Russia; Institute of Cytology and Genetics of the Siberian Branch of the Russian Academy of Sciences, Novosibirsk, Russia Kurchatov Genomic Center of ICG SB RAS, Novosibirsk, Russia Novosibirsk State University, Novosibirsk, Russia

**Keywords:** management, LIMS, ELN, version control systemsFAIR, Trello, GitHub, Redmine, SEEK, OpenBIS, Galaxy, управление, LIMS, ELN, FAIR, системы контроля версий, Trello, GitHub, Redmine, SEEK, OpenBIS, Galaxy

## Abstract

Modern investigations in biology often require the efforts of one or more groups of researchers. Often these are groups of specialists from various scientific fields who generate and share data of different formats and sizes. Without modern approaches to work automation and data versioning (where data from different collaborators are stored at different points in time), teamwork quickly devolves into unmanageable confusion. In this review, we present a number of information systems designed to solve these problems. Their application to the organization of scientific activity helps to manage the flow of actions and data, allowing all participants to work with relevant information and solving the issue of reproducibility of both experimental and computational results. The article describes methods for organizing data flows within a team, principles for organizing metadata and ontologies. The information systems Trello, Git, Redmine, SEEK, OpenBIS and Galaxy are considered. Their functionality and scope of use are described. Before using any tools, it is important to understand the purpose of implementation, to define the set of tasks they should solve, and, based on this, to formulate requirements and finally to monitor the application of recommendations in the field. The tasks of creating a framework of ontologies, metadata, data warehousing schemas and software systems are key for a team that has decided to undertake work to automate data circulation. It is not always possible to implement such systems in their entirety, but one should still strive to do so through a step-by-step introduction of principles for organizing data and tasks with the mastery of individual software tools. It is worth noting that Trello, Git, and Redmine are easier to use, customize, and support for small research groups. At the same time, SEEK, OpenBIS, and Galaxy are more specific and their use is advisable if the capabilities of simple systems are no longer sufficient.

## Abstract (ru)

Современная исследовательская работа в биологии нередко требует усилий одной или нескольких групп исследователей. Часто это группы специалистов из смежных областей, которые генерируют и обмениваются данными разных форматов и размеров. Без применения современных подходов автоматизации работы и версионирования данных (когда данные от разных сотрудников сохраняются в разные моменты времени) коллективная работа быстро переходит в неуправляемый хаос. В настоящем обзоре приведен ряд информационных систем, предназначенных для решения озвученных задач. Их применение для организации научной деятельности позволяет управлять потоком действий и данных, добиваясь работы всех участников с актуальной информацией, и решением вопроса воспроизводимости как экспериментальных, так и вычислительных результатов. Описаны методики по организации потоков данных в рамках работы коллектива, принципы по организации метаданных и онтологий. Рассмотрены информационные системы Trello, Git, Redmine, SEEK, OpenBIS и Galaxy. Описана их функциональность и сфера использования. Выбирая те или иные инструменты, важно понимать цель внедрения, определить набор задач, которые они должны решать, и исходя из этого формулировать требования и отслеживать применение рекомендаций на местах. Задачи по созданию структуры онтологий, метаданных, схем хранения данных и программных систем являются ключевыми для коллектива, который решился на проведение работ по автоматизации оборота данных. Не всегда возможно внедрить такие системы целиком, но все же следует стремиться к этому через поэтапное внедрение принципов по организации данных и задач с освоением отдельных программных инструментов. Следует отметить, что системы Trello, Git и Redmine проще в использовании, настройке и поддержке для малых исследовательских групп. В то же время SEEK, OpenBIS и Galaxy более специфичные, их применение целесообразно в случае, если возможностей простых систем уже недостаточно.

## Introduction

Modern research work in biology often requires the efforts of one or more groups of researchers. Often, these are groups of specialists from related fields who generate and exchange data of different formats and sizes. To automate and computerize this work, various tools are used to catalog, log the progress of experiments, and record results: paper notebooks and laboratory journals, spreadsheet programs, and report writing in various text editors. Without the use of modern approaches of work automation and data versioning, the team quickly succumbs to “uncontrollable chaos”. Acritical point in the organization of interaction in the team is the complexity of the procedure of knowledge transfer from one team member to another, as such knowledge is not formalized and often contains notes understandable only to the author. All this leads to delays in the next stages of research or in the design of publications. Sometimes employees forget to record new facts and notes, or do not keep any records of intermediate stages of work at all. This leads to irretrievable loss of knowledge and waste of resources for repeated experiments and observations.

When collecting primary data, researchers may also make errors in processing values or assigning them to a particular category. For example, transcriptome data may be erroneously attributed to a different organism from the one from which it was obtained; data may not be recorded in a uniform manner, using values of different types (integer, floating point number, string, date, etc.). Also, when working with Excel, strings may be mistakenly converted to floating point numbers, which can be critical to the interpretation of the study results (Zeeberg et al., 2004), so implicit data conversions should be avoided. In (Roche et al., 2015), Bioresource Collections (BRCs) in Ecology and Evolution were analyzed. It was found that 56 % of these BRCs were incomplete, i. e. there were blank values in the tabular data, and 64 % were collected in such a way that it was not possible to reuse the stored data due to errors in recording values.

Therefore, every team faces the task of properly formalizing the processes of data management and knowledge sharing between employees. In the following article, we will look at specific data organization methodologies and information systems and software tools that implement them, which are used by scientific organizations to distribute tasks and automate the flow of work data.

## Data and process organization methodologies

There are several ways to address the challenge of organizing scientific data flows, but all require the research team to create systems of arrangements for managing, processing, and communicating scientific information. Automation systems with managed access help preserve knowledge, regulations and other “substances” of laboratory work, and do not require constant coordination. The following issues arise at the outset of these activities: (1) use of existing data design standards developed by the professional community; (2) formalization or creation of a common “working language” within the team; (3) deployment, implementation and maintenance of the information system and setting up access rights for user groups.

Transition to the use of existing standards and formats for data representation or the creation of one’s own formats with comprehensive documentation sufficient for unambiguous interpretation of values allows to overcome the problem of knowledge transfer between employees inside and outside the team. Supporting documentation will be used to automate work with the information system, for example, to build modules for generating summary diagrams and reports. Formal schemes for describing the results of scientific activity have recently become widely used for fast information retrieval and interpretation of these files not only by machines but also by people. Examples include mathematical models in SBML (Hucka et al., 2019), SBGN (Novère et al., 2009) formats supported by the CO.MBINE community (Schreiber et al., 2015). We also note the MIRIAM approach for describing holistic biochemical systems (Novère et al., 2005) and the MIAME format (Brazma et al., 2001) for describing sequencing results on microarrays or RNA sequences

When data representation standards are defined, the stage comes to formalize or create a common working language and exchange protocols within the team to streamline the transfer of subject matter knowledge. If we leave the “as it is convenient/as it was before” approach to data presentation, the issue of ambiguous or missing knowledge in the database will not be solved, which will lead to additional resource costs for correcting data at later stages of work. Ontology tools (Guizzardi, 2020) can help in solving the problem of formalization and creating a common working language. Ontologies are a broader class of knowledge organization systems for describing results in comparison to the aforementioned formal schemas. In ontology systems, it is possible to establish “concepts” and “relations” between concepts, rather than strictly follow a ready-made schema proposed by someone earlier. Ontologies are created in order to describe meaningful information and to unambiguously interpret a system of concepts and processes within and outside the team. Teams use both simple methods to describe ontologies, such as first-order logic language, and more complex tree structures, such as OntoUML (Guizzardi et al., 2018) or RDF schemas (Gutierrez et al., 2007). Also, mathematical category theory (Kuś, Skowron, 2019) is gaining popularity for composing ontological relationships of a subject domain, which is designed to connect different areas of mathematics and subject domains with each other. A graphical language of “ontology logs” (English “ologs”, essentially descriptions of a subject area in the form of graphs, where nodes describe objects with certain properties and edges describe functions of transforming one object into another) has also been implemented using the foundations of this theory (Spivak, Kent, 2012). Currently, the tools and language of category theory are not widely used in scientific publications and systems, but there are works on the use of this language in neurobiology (Brown, Porter, 2008) and on the mathematical description of an evolving model of memory (Ehresmann, Vanbremeersch, 2007).

One way to formalize the stages of laboratory work is to create metadata – information describing the data themselves (Roche et al., 2015). The format of their description is rather free. Metadata can be described/represented in the form of a structured file (XML or JSON) or database tables of both relational (Postgrespro.ru) and document-oriented structure (MongoDB.com). The description can contain any information, such as what the columns in the tables mean, what units of measurement are used, from which organism the materials were collected, how these results were obtained. Metadata can be used in conjunction with ontology systems and formal schemes for representing scientific results for quick retrieval of relevant information and unambiguous interpretation of results.

The FAIR research community (Wilkinson et al., 2016) proposed their set of principles for describing data and metadata in the task of storing and transferring information both between teams of researchers and between different data analysis programs. They formulated the following four principles that a laboratory information system should possess:

1. Findable – (meta)data are unique and uniquely identifiable. The system should have a basic mechanism for reading a detailed description, and should be able to search for these data by key fields. 2. Accessible – the data are readable by both humans and computers for further work. This is achieved using standard formats and protocols. 3. Interoperable – (meta)data are described in a machinereadable form, in a usable format and are annotated using ontologies. 4. Reusable – (meta)data are sufficiently well described so that they can be shared with other people and systems for further analysis. This item is a logical consequence of the above items.

Next, let’s look at software tools that are worth considering for solving data management challenges on the path to research automation.

## Software tools

Two concepts, LIMS and ELN (Barillari et al., 2016), which are implemented in software packages for research control tasks, are shown in the Figure.

**Fig. 1.**
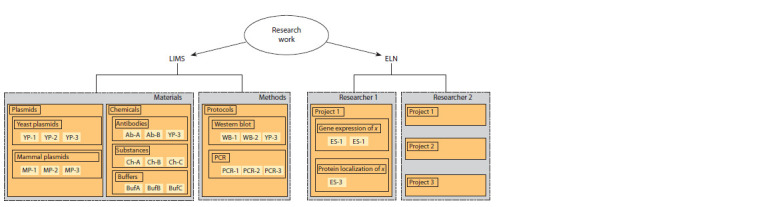
Description of the data structure stored in LIMS and ELN systems

LIMS (Laboratory Information Management System) is a laboratory information management system. Its tasks include the management and control of laboratory materials and methods. With the help of this system, researchers can carry out document management with administration, and companies, create schedules for the use of instruments, record reagents, research objects, etc.

ELN (Electronic Laboratory Notebook) is an electronic laboratory journal. The tasks of such systems include management of projects, experiments, users, research groups, as well as logging (journaling) and control of experiments. In essence, these systems replace the functions of paper notebooks for entering and transmitting notes as experiments progress.

## Trello

Trello (https://trello.com/) is a conditionally free web-based workflow and communication service. In this system, users set up a virtual whiteboard on which “cards” with “tasks” are placed. The board itself is divided into sections, between which the cards are moved, showing the movement through the work stages. Most often, the board sections are marked with the statuses of work execution, for example, “tasks in queue”, “in progress”, “waiting for feedback”, “task completed”. It is possible to independently create sections according to your own scenario, which best reflects the workflow of the team. In this way, employees and managers can: (1) monitor the progress of work in real time; (2) change the statuses of tasks, add comments to tasks; (3) link tasks to each other; (4) react at early stages in cases of suspended work.

The disadvantages of Trello include the inability to modify the functionality of the system with its own modules and limited functionality in the free version. The analogs include Yandex.Tracker (https://cloud.yandex.ru/services/ tracker), GutHub Projects (https://docs.github.com/en/issues/ planning-and-tracking-with-projects/learning-about-projects/ quickstart-for-projects) and Kanboard (https://kanboard.org/). The proposed tools are focused on the implementation of ELN requirements, but users can adapt them to LIMS tasks. They are aimed at managing team processes – other tools should be used to organize the storage and movement of the data themselves.

## GitHub

When a team works together on program codes, documents and reports, there is an important task of change control. Mail clients and person-to-person networking do not cope well with this task, as the users themselves need to control the relevance of the versions of these documents. Also, the task of versioning data and text is not solved due to the lack of a system for centralizing the storage of files and fixing their changes. It is these tasks that can be solved by using the Git program (Chacon, Straub, 2014).

The Git program creates repository files in a local folder, allowing you to navigate between changes to the files. This system is most often used by programmers to work on a project simultaneously, comparing and merging code changes from different developers. Open source projects are most often stored publicly on the GitHub project servers (https://github. com). Some research groups use the Git version control system to produce articles and dissertations. For example, it was used to write a mathematical book on homotopy type theory (The Univalent Foundations Program, 2013). About 20 people worked on the book, and the cloud storage service Dropbox could not cope with the task of synchronizing the text. As a result, the team produced a 600-page book in less than six months (https://math.andrej.com/2013/06/20/ the-hott-book/).

GitHub itself cannot be installed on a local computer, but there are similar solutions that can be installed on a local system, such as GitLab (https://gitlab.com), Gogs (https://gogs.io), Gitea (https://gitea.com), and GitWeb (https://git-scm.com/docs/gitweb). Within these systems, it is possible to solve ELN and LIMS tasks, but users will have to understand Git in detail.

## Redmine

Redmine (https://redmine.org/) is widely used as a project control and task assignment system. Most often, the main project manager (administrator, head of laboratory, etc.) creates a set of tasks and assigns responsible executors. The executors change the status of task readiness as they complete the task. The system automatically monitors the status of project tasks and builds summary diagrams that show the time discrepancy between the plan and the actual execution. Also, the main functions of this system include:

• Role creation and restriction – the administrator can create several additional user roles and set rules for them to work in the system (reading and/or writing “tasks”, wiki pages and so on). • Flexible error control system – the function is widely used in software development, when testers or users add a “task” of the “error” type to the system to notify developers. • Calendar and Gantt Chart. They are used to keep track of task due dates. • Adding project news with notification of participants. • Adding documents and files to the system. • Notifying users by e-mail or RSS feed. • Formalization of knowledge for each project in the format of Wikipedia – an electronic encyclopedia/reference book in the form of Internet pages. • Forum system for each project – the ability to publicly discuss in one place the solution of problems; the ability to quickly run your eyes over the chains of messages on the topic. • Time accounting of work on tasks and the project as a whole. • Creation of user forms and fields for additional description of “tasks”, “projects”, “users” and other entities within this system.

The system can be deployed in a local information environment (up to a personal computer). It is possible to add new functionality through the implementation of submodules (plug-ins). The disadvantages of Redmine include the absence of a task board like Trello, which is clear and easy to use, as well as the limited functionality of the standard version. Therefore, for full-fledged work, it is necessary to install third-party submodules.

Many teams in the IT sector have built their workflows on the basis of the Redmine software system. In 2019, the ENVRI-FAIR project (Petzold et al., 2019) was launched to connect resources and data between the European Environmental Research Infrastructure (ENVRI) cluster and the European Open Science Computing Cloud (EOSC) using Redmine (this information was obtained from the technical documentation of this project). Based on Redmine, it is possible to realize the solution of both ELN and LIMS tasks.

## SEEK system

The SEEK system (Wolstencroft et al., 2015) is designed to manage, disseminate, and explore mathematical models and associated systems biology data. SEEK organizes research project information including experimental data and bioinformatics results within a structure of three entities: Investigation, Stage, Assay (ISA) (Rocca-Serra et al., 2010). Investigation reveals the essence of a particular project (who is doing the work, which institute, the time of the study). Stage describes a specific stage in the course of the study (excretion of DNA or protein from the tissue of the organism under study, mapping of RNA reads to a reference genome, etc.). Assay is the unit of the result of the work performed. Also, in the system, it is possible to establish associative connection between samples

The advantage of this system is the linking of data with each other within the above structure with the description of the research team, as well as the reformatting of metadata into an RDF knowledge graph (Gutierrez et al., 2007) using Virtuoso server (Software, 2022). Metadata are described mainly in tabular form (ISA-Tab), there is also a possibility to use JSON schema. For manual annotation of data, SEEK developers suggest using FightField software. Search of RDF graph data using SPARQL query language is flexible in use – in comparison to SQL, where, in addition to writing data selection rules, the user is required to manually describe the list of tables and the way they are joined. Another problem with SQL is that the user has to optimize their queries to perform searches quickly.

The main focus of SEEK is the storage and transfer of mathematical models of biological processes, the resource also allows working with SBML models and opening them in JWS Online (Olivier, Snoep, 2004) and in COPASI (Hoops et al., 2006). This system mainly implements the ELN requirements for bioinformatics projects and LIMS is not implemented in it.

## OpenBIS system

As part of the laboratory work, researchers are tasked with creating protocols of experiments, following these protocols and fixing the results of work, fixing events, etc. There is a need to align the results of a series of works within a single project, for example, linking experiments to different organisms, their phenotypes, genotypes, developmental environment and other data. OpenBIS (Bauch et al., 2011) provides functionality to store and align metadata under detailed descriptions of experiments, their results, parameters, etc. The OpenBIS system consists of three modules: application server, data server and metadata database.

• The application server is the access point for users. The module provides access to the program complex through a graphical user interface, as well as via HTTP protocol (OpenBIS provides libraries in the Python, Java and Matlab programming languages for interaction over the network). To add new functions (e. g., mass spectrometry data storage), OpenBIS provides a system of modules, each of which must be implemented in the Jython programming language. This module divides authorization among users (read data, read/write data).

• The data server performs the work of organizing primary data storage on disk drives. • The metadata database is a PostgreSQL database management system (DBMS). This module links data in projects, stores metadata, points to data from the data server, provides data search tasks. • The ability to link to data on external resources (BigDataLink module). Metadata are stored in the metadata base, while the original information is not stored on the data server, but remains on external resources. This function is used when working with large files. • Extension of functionality using libraries in Java, Python, JavaScript, Matlab for interaction with the OpenBIS system (data retrieval/downloading, metadata search). These libraries use hardware interface REST API of OpenBIS service, so it is possible to realize modules for interaction with the system in other programming languages. It can be used for realization of automated calculations with attraction of stored data from the OpenBIS system. • The structure of data storage is hierarchical and organized as follows: space, project, experiment/collection, Object/ Sample, Data Set. • To link objects and data with each other, there is a method to establish ancestor–descendant relationships, i. e. the system can create a graph of objects and data. • Import/export of data in tabular form. • Realization of additional functionality of the system itself by means of a system of modules. • The system performs audit of each change in its databases. • Semantic annotation of data – description of results in a convenient and interpretable format. An RDF schema (Gutierrez et al., 2007) is used to describe the semantics. • Integration with the SEEK system.

This system has proven itself for primary storage of biological information obtained during experiments. In (Friedrich et al., 2015), a system was implemented to add and record experimental data on different tissues of organisms when different drugs were administered. At the first level of the storage system, the object of study is described (e. g., a particular mouse in the laboratory that has been injected with a particular drug). The second level describes the particular biological tissue that was extracted from the subject. The third level describes the sequences (nucleotide or protein sequences) extracted from the object tissue under study. The system is based on LIMS and ELN requirements and is an exemplary implementation of them.

## Galaxy

The systems described above are mainly systems for controlling laboratory data, but the challenges for bioinformatics laboratories are exactly the same: control of data flow, reproducibility of calculations, access to data and their storage in the server. The Galaxy system (Galaxy Community, 2022) has been implemented to solve such problems. Galaxy consists of the following modules: (1) a server with a software and GUI interface and (2) workflows, which run analytic pipelines at the request of users. Users can either independently run the programs installed in the server and store their data (sequences, annotations, protein lists, etc.) there.

Computational pipelines can be implemented in the form of a graph, where the vertices denote programs with configured parameters, and they are connected by edges that denote the direction of data movement from the output of one program to the input of another. These processes can also run programs on a remote server or cluster, and exchange files through a common file system. Reproducibility of computational programs is achieved using the Conda environment system (Yan Y., Yan J., 2018), where an independent environment (a set of libraries, programs, and modules in Python\R of strictly defined versions) is created for each program. The lightweight virtualization system Docker (Rad et al., 2017) can also be used, where the program runs in a “virtual” and “lightweight” operating system of the Linux family. Galaxy is a FAIR-like system (Hiltemann et al., 2023). In essence, Galaxy implements an ELN requirements system but in the domain of bioinformatics pipelines, i. e., it is not a full ELN. LIMS is not fully implemented, there is only multi-user input and a limitation on the storage of computational results.

## Conclusion

In this paper, a limited number of information solutions in the field of organization of project activities of laboratories working in the field of biology were considered. The Table describes brief characteristics of the reviewed systems. Such solutions as OpenBIS, SEEK and Galaxy were created specifically for scientific work, while Trello and Redmine are project management systems of more general categories, although they can be used in the work of scientific groups. The Git software suite can be considered by large teams as a tool for sharing and versioning program code, data, article texts, monographs, and other scientific texts. It should be noted that Git is not intended for storing binary files (in particular, files in DOCX, PDF, etc.), as it only considers changes to text files. Markdown and LaTeX are more appropriate formats for this use of Git.

**Table 1.**
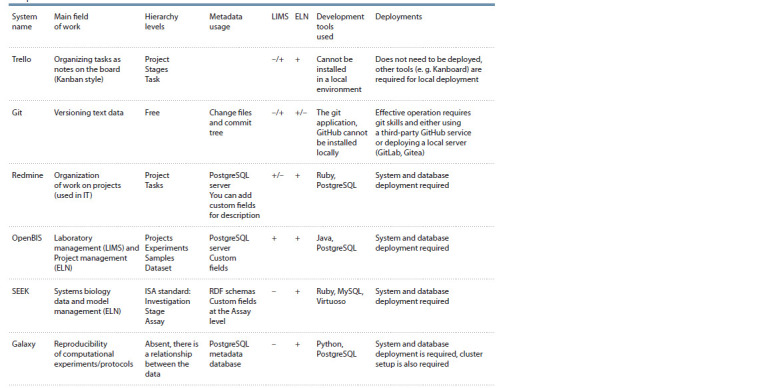
Comparison of software solutions

Before implementing these or those tools, it is important to understand the goals of their implementation; based on the goals, it is important to formulate requirements, define a set of tasks to be solved by the system, and monitor the application of recommendations by specific implementers. Taking into account the complexity of the above processes, it can be recommended to start with implementation from open formats and standards for presentation and transmission of biological data proposed and developed by the scientific community. The use of general-purpose workflow systems in the laboratory will allow obtaining operational experience, which, in turn, will help to determine the data formats, work protocols, and software products required for the laboratory, and, based on this, to make a decision on scaling the automation of work with data, including the creation of ontology structures, metadata, storage schemes, and scenarios for the operation of software systems.

## Conflict of interest

The authors declare no conflict of interest.
